# Occurrence and function of enzymes for lignocellulose degradation in commercial *Agaricus bisporus* cultivation

**DOI:** 10.1007/s00253-017-8294-5

**Published:** 2017-05-02

**Authors:** Mirjam A. Kabel, Edita Jurak, Miia R. Mäkelä, Ronald P. de Vries

**Affiliations:** 1 Laboratory of Food Chemistry, Wageningen University, Bornse Weilanden 9, 6708 WG Wageningen, The Netherlands; 2 Department of Biotechnology and Chemical Technology, Aalto University, Kemistintie 1, 02150 Espoo, Finland; 3 Fungal Physiology, Westerdijk Fungal Biodiversity Institute & Fungal Molecular Physiology, Utrecht University, Uppsalalaan 8, 3584 CT Utrecht, The Netherlands; 4 Division of Microbiology and Biotechnology, Department of Food and Environmental Sciences, University of Helsinki, Viikinkaari 9, Helsinki, Finland

**Keywords:** *Agaricus bisporus*, Xylan structure, Lignin, Genome, Enzymes

## Abstract

The white button mushroom *Agaricus bisporus* is economically the most important commercially produced edible fungus. It is grown on carbon- and nitrogen-rich substrates, such as composted cereal straw and animal manure. The commercial mushroom production process is usually performed in buildings or tunnels under highly controlled environmental conditions. In nature, the basidiomycete *A. bisporus* has a significant impact on the carbon cycle in terrestrial ecosystems as a saprotrophic decayer of leaf litter. In this mini-review, the fate of the compost plant cell wall structures, xylan, cellulose and lignin, is discussed. A comparison is made from the structural changes observed to the occurrence and function of enzymes for lignocellulose degradation present, with a special focus on the extracellular enzymes produced by *A. bisporus*. In addition, recent advancements in whole genome level molecular studies in various growth stages of *A. bisporus* in compost are reviewed.

## Introduction

Edible mushrooms are an important agricultural product worldwide. Only few of these edible mushrooms, however, can be cultivated with the most extensively cultivated species being *Agaricus bisporus* (30–40%), *Pleurotus ostreatus* (25–27%), *Lentinula edodes* (17%) or *Volvariella volvacea* (16%) (Chang 1999; ISMS Edible mushrooms 2017; Royse 2014). The white button mushroom *A. bisporus* can be grown on various raw materials, such as composted cereal straw and animal manure. This cultivation process is usually conducted in buildings or tunnels where the environmental conditions, such as temperature, humidity and concentration of carbon dioxide, are controlled. The major regions of *A. bisporus* cultivation are Europe, North America, China and Australasia.

**Fig. 1:**
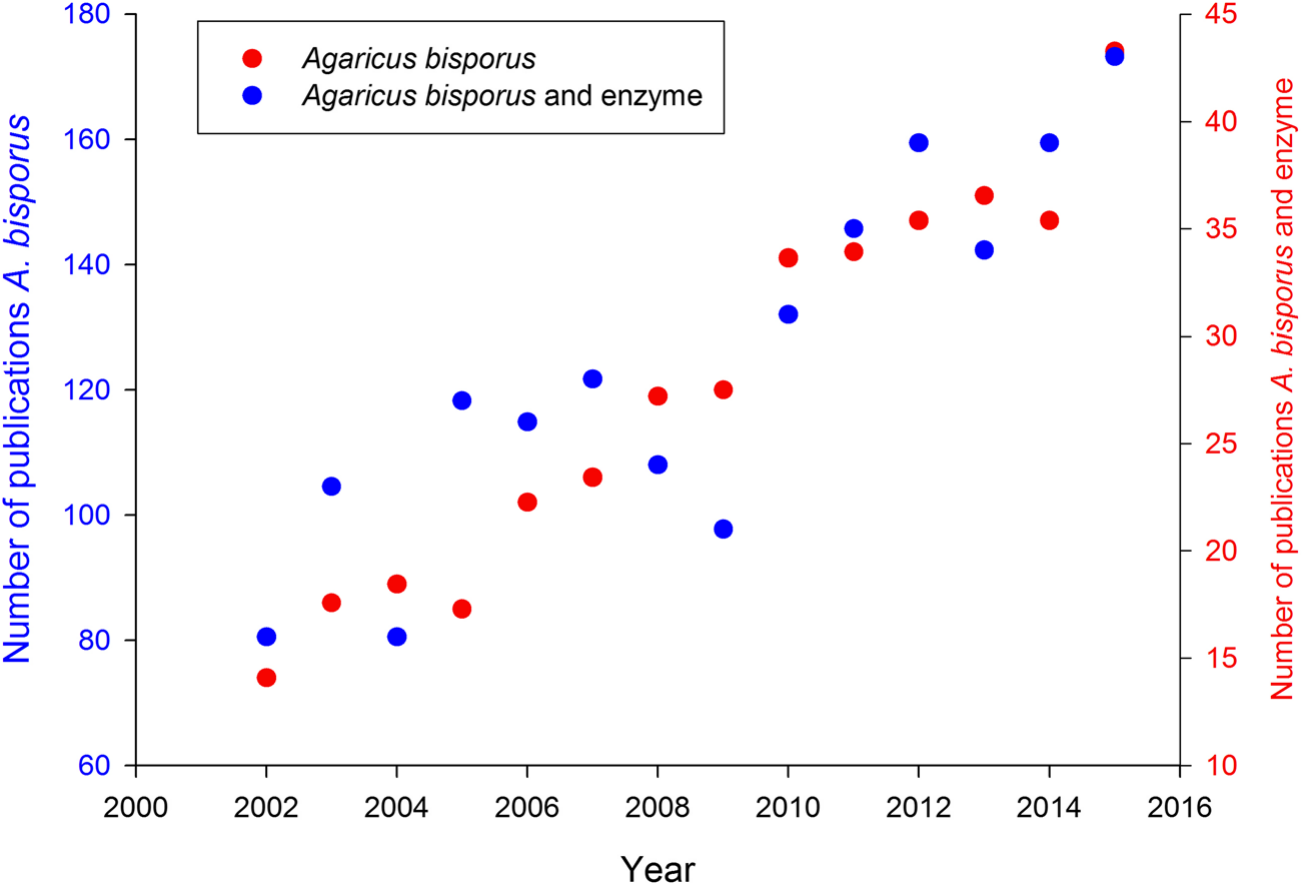
Scientific publications (Web of Science) per year of the topic *Agaricus bisporus* or *Agaricus bisporus* and enzyme

Besides its commercial importance, the basidiomycete *A. bisporus* has a natural life style as a saprotrophic leaf litter (non-wood)-inhabiting decayer of plant biomass and, hence, contributes to the carbon cycle in terrestrial ecosystems (Morin et al. 2012). It has a widespread geographical distribution in natural habitats such as arid places or forests in North-America and forests or coastal dunes in Europe or Africa (Geml et al. 2008; Kerrigan 1995; Callac et al. 2002). The life cycle of *A. bisporus* consists of a vegetative mycelial phase with a subsequent reproductive phase in which fruiting bodies are formed. Vegetative mycelium, generally, supplies nutrients for the growth of fruiting bodies, while the role of fruiting bodies is reproduction (Bonner et al. 1956). The role of enzymes secreted by *A. bisporus* during either vegetative or reproductive phases has received a steady interest (Fig. 1) but has not been reviewed so far.

In this mini-review, the occurrence and function of enzymes for lignocellulose degradation reported for commercial mushroom cultivation are discussed, focussing in particular on the extracellular enzymes of *A. bisporus*. Enzyme activity and protein occurrence are compared with recent published advancements in understanding the genetic potential of *A. bisporus* and the gene expression in both vegetative and reproductive phases. A correlation between the produced enzymes and the fate of lignocellulose structures during *A. bisporus* growth is also presented. We conclude with an outlook on how the scientific insights provided in this mini-review can help to improve commercial cultivation of mushrooms.

## Commercial cultivation of *Agaricus bisporus*

**Fig. 2:**
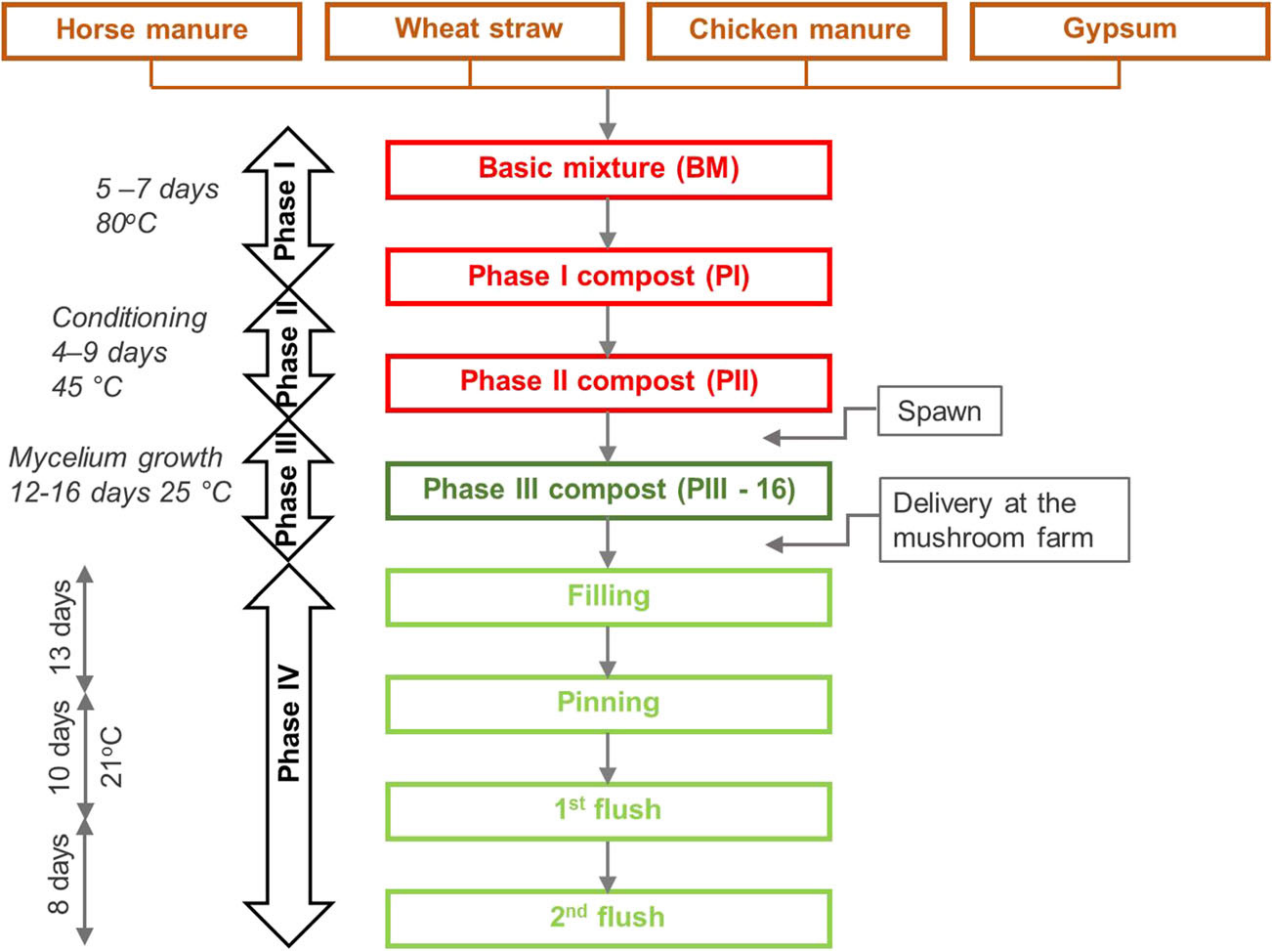
Schematic overview of the composting and *A. bisporus* mushroom growth process. At the end of the first and second flush, the fruiting bodies are harvested

The production of compost in commercial production facilities comprises the bioconversion of raw materials into a substrate supporting the growth of *A. bisporus*. The whole process, from composting to fungal mycelium growth and production of fruiting bodies, has been optimized over the last century. A schematic overview of the process is shown in Fig. 2. Process optimization was based on empirical approaches, while a full understanding of the degradation and conversion pathways at the various conditions performed was not considered. Now, the mushroom industry more and more believes that the next improvements in their process will result from a more detailed understanding of the biological mechanisms and metabolic pathways involved in the production process. Hence, the traditional craft of mushroom production for food, from agricultural by-products and manure, becomes a true science.

World-wide, the type of raw materials used to produce a good substrate for *A. bisporus* growth varies, although they always contribute as a carbon and a nitrogen source (Iiyama et al. 1994). In Europe, the *A. bisporus* substrate or compost is produced from a mixture of wheat straw (40 to 50% of the total dry weight), horse manure or stable bedding (20–25%), poultry manure (10–15%) and gypsum (5 to 10%). Horse manure or stable bedding is only used in certain countries where these ingredients are available in significant amounts.

The production of the *A. bisporus* substrate is usually performed in two indoor phases (Fig. 2): Phase I (PI) and Phase II (PII). At the start of PI, raw materials are mixed and, typically, mesophilic microbiota starts to develop. This microbiota converts part of the carbohydrates and proteins into heat and ammonia. As the temperature rises, the mesophilic microbiota is naturally replaced by thermophilic microbiota (Gerrits 1988). In PI, the compost temperature rises up to 80 °C and could last 3 to 7 days. These reactions cause the wheat straw to soften. During PII, the compost is conditioned (8 h at 56 °C), partly by blowing air and partly by further growth of microbiota, and kept at 45 °C until the process air is virtually free of ammonia (Gerrits 1988). In this phase, microorganisms, in particular actinomycetes and fungi, are reported to grow, which consume part of the ammonia (about 40%) (Gerrits 1988). In PII, the thermophilic ascomycete fungus *Humicola insolens* var. *thermoidea* (*Scytalidium thermophilum*) could be found in the compost. This fungus is reported to increase the growth rate of the *A. bisporus* fruiting bodies during later stages of the production process (Fermor and Grant 1985; Straatsma et al. 1989). Final PII compost has been shown to consist mainly of lignocellulosic components from wheat straw together with microbial biomass (Eddy and Jacobs 1976; Martínez et al. 2008).

At the end of PII, the compost is inoculated with millet or rye grains colonized with *A. bisporus* mycelium, which grows through the compost until after 12 to 16 days, and the compost is fully colonized and considered mature (Fig. 2). The latter phase is named Phase III (PIII; Fig. 2). Typical temperatures for this stage are 20–25 °C. The mycelium of *A. bisporus* has been reported to not only consume most of the microflora present in the compost (Fermor and Wood 1981) but also cellulose, hemicellulose and lignin or humic-like compounds (Durrant et al. 1991). The mature compost with an additional top casing layer is ready for fruiting body formation in Phase IV (PIV; Gerrits 1988; Fig. 2). After 18 to 21 days, the first harvestable mushrooms could appear and after that in repeating 7- to 8-day cycles known as flushes (Flegg and Wood 1985; Fig. 2).

## Genome potential of *A. bisporus* for lignocellulose degradation

The genome sequence of *A. bisporus* was published in 2012 (Morin et al. 2012) and revealed clear adaptation of this species to its natural humic-rich biotope. It has a full repertoire of plant polysaccharide degrading enzymes, but particularly notable was the expansion of heme-thiolate peroxidases (HTPs) and β-etherases, compared to wood-degrading species of the Agaricomycotina. The corresponding genes were upregulated during growth on humic-rich compounds together with other lignin and humic compound-related genes, such as those encoding manganese peroxidases (MnPs), copper radical oxidases and cytochrome P450s (Morin et al. 2012).

A more detailed analysis of the expression of carbohydrate active enzyme (CAZyme) encoding genes in compost casing layer and fruiting body during commercial cultivation of *A. bisporus* revealed a clear tissue-type related regulatory system (Patyshakuliyeva et al. 2013). Compost grown mycelium expressed a large diversity of CAZy genes related to the degradation of plant biomass components, while fruiting bodies mainly expressed CAZy genes related to synthesis and modification of the fungal cell wall. An intermediate profile reflected the morphological change that occurs in the casing layer. These differences were also visible at the metabolic level as the compost-grown mycelium-expressed genes of a wide variety of sugar catabolic pathways, while in the fruiting body, only glycolysis-related genes were expressed (Patyshakuliyeva et al. 2013). This demonstrated that the diversity of sugars released by the CAZymes is being converted simultaneously by *A. bisporus*, but in fruiting bodies apparently only glucose, glucose derivatives, such as sorbitol or trehalose, and mannitol are converted into fungal biomass. Accumulation of other monosaccharides or other sugar-alcohols could not be detected in the fruiting bodies, thus implying that only these compounds are transported into the fruiting body from the mycelium. This suggests that sugar transport to the fruiting body is not solely an osmotically driven process but involves either specific transporters or carrier proteins.

More recently, a detailed comparison of the plant polysaccharide degrading potential of a large set of basidiomycetes was presented (Rytioja et al. 2014). This set included mainly not only white rot and brown rot species but also several litter/straw-degrading species as well as some ectomycorrhizal and plant pathogenic species. Three of the litter/straw-degrading species (*A. bisporus*, *Volvariella volvacea* and *Coprinopsis cinerea*) are highly similar in their CAZy content, while significant differences in gene numbers could be found in *Galerina marginata* for several CAZy families. While these species are taxonomically related to ectomycorrhizal species, their CAZy genome content is more similar to white rot species, likely driven by the requirements of their ecological niche (Rytioja et al. 2014). Interestingly, there is very little difference in the genome content of *A. bisporus* var. *bisporus* and *A. bisporus* var. *burnettii* (Morin et al. 2012, Rytioja et al. 2014), despite the first being used for commercial mushroom cultivation on compost, while the second is an ecological isolate from leaf litter.

## Fate of lignin and substituted xylan correlated to *A. bisporus* gene expression and enzyme activities

### Composting phases I and II—changes in plant cell wall structures of compost

The main carbon source for *A. bisporus* in compost could arise from grass-like materials, such as wheat straw used in e.g. Europe. In general, wheat straw is composed of the cell wall polysaccharides cellulose and xylan and of the cell wall polymer lignin. For completeness, briefly, the structural changes in compost in the composting phases PI and PII (Fig. 2) described in literature are discussed. In these two phases, the changes are provoked by microbial growth other than *A. bisporus*, which is not yet introduced to the compost. In the first composting phase (PI), the wheat straw carbohydrates, cellulose and xylan, were only metabolized to a limited degree. Jurak et al. (2015a) found that in PI, less than 10% of the carbohydrates were consumed in a mass balance experiment. Furthermore, no changes in compost composition were observed, but visually, the compost became softer. In contrast, in Phase II, up to 50% of the compost carbohydrates were metabolized, mainly by bacteria and some fungi other than *A. bisporus* (Jurak et al. 2015a). At the same time, separation along the middle lamella between plant cells of wheat straw has been observed and, hence, a decrease in rigidity of the connected cells (Atkey and Wood 1983). After these two phases (PI and PII), the compost is considered ready for *A. bisporus* colonization (Fig. 2). At this point, the compost is shown to be composed of around 26% (*w*/*w*) of carbohydrates, of which half is xylan and half is cellulose, 26% (*w*/*w*) of lignin, 31% (*w*/*w*) of ash and around 10% (*w*/*w*) of protein (Jurak et al. 2014).

### Vegetative mycelium growth—changes in plant cell wall structures of compost

During mycelium growth in PIII (Fig. 2), less than 6% of xylan and no cellulose were metabolized (Jurak et al. 2015a). Relatively low enzyme activities targeting xylan, arabinan, galactan, mannan and xyloglucan were present in the compost extracts from spawning stage day 16 (PIII) (Jurak et al. 2015b). In contrast, 40% of lignin was metabolized (Jurak et al. 2015a). In the latter study, lignin was analysed by pyrolysis GC-MS (Py-GC-MS) and with the gravimetric ‘Klason’ lignin analysis. Both methods do not distinct between native lignin and partly degraded or modified lignin-like structures. Hence, it cannot be concluded if only native lignin was metabolized or also previously modified lignin-like or humic-like structures (Smith 1994). Although only a limited amount of xylan was metabolized, about 20% of the total remaining xylan in the compost became water soluble, compared to 3% in compost at the start of PIII. In addition, PIII-alkali extracted xylan showed a decreased molecular weight (Jurak et al. 2014). Possibly, xylanases present, although their activity was reported to be low, partly depolymerized xylan structures in the compost. Albeit xylan was degraded, the carbohydrate composition, and the degree of substitution of the xylans in compost at the beginning and end of PIII was rather similar (Jurak et al. 2014).

At the end of PIII, lignin or lignin/humic-like structures were not only metabolized for 40%, also, the remaining lignin was reported to show a different fingerprint compared to earlier stage composts, as analysed by Py-GC-MS. In summary, the ratio between syringyl-like and guaiacyl-like units (S/G ratio) increased from 0.5 to 0.7 during PIII. Furthermore, the ratios of Py-GC-MS analysed vinyl-substituted guaiacol to guaiacol and vinyl-syringol to syringol decreased during PIII (Jurak et al. 2015a). Previously, studies on lignin degrading white rot fungi have shown that the extracellular oxidative enzymatic machinery involved in lignin degradation is induced by starvation (Ten Have and Teunissen 2001). Therefore, it can be postulated that due to the conversion of 50% of carbohydrates in the composting stages prior to PIII, only carbohydrates that are more difficult to degrade remain in the compost at the start of PIII. As a result, the lignin degrading machinery of *A. bisporus* is induced in Phase III, resulting in the lignin degradation and modification observed. This is supported by the highest level of expression of the lignin-modifying enzymes encoding genes of *A. bisporus* detected during spawning stage day 16 (PIII) compared to all the other composting phases where no significant expression was observed (Patyshakuliyeva et al. 2015). These included genes encoding two laccases (AA1_1), one MnP (AA2), two glucose-methanol-choline (GMC) oxidoreductases (AA3_2), four glyoxal oxidases (AA5_1) and one 1,4-benzoquinone reductase (AA6). Of the ligninolytic genes and enzymes, laccases were expressed and produced at the highest level in the compost. Laccase has already been identified by Wood (1980) as major product of protein synthesis during mycelial growth of *A. bisporus*. Wood reported that laccase comprised 0.7% of the fungal biomass or 2.1% of fungal protein, assuming that 33% of the biomass dry weight was protein. This laccase amount was based on the specific activity measured for purified laccase and indicates the importance of laccase for the nutrition of *A. bisporus* (Wood 1980).

Lignin composition remained unmodified after the spawning stage day 16 until the stage when the second flush was completely harvested, suggesting that sufficient carbohydrates have become accessible during the PIII phase. In grasses, such as wheat straw, the amount of lignin in the plant cell wall is often negatively correlated to the digestibility of the cell wall carbohydrates (Grabber et al. 2004). This supports the hypothesis that the decrease and changes in lignin in PIII, during the growth of *A. bisporus* mycelium, improve the digestibility of carbohydrates in the later growth phases.

The *A. bisporus* genome encodes a limited repertoire of lignin-modifying enzymes compared to wood-decaying white rot species (Morin et al. 2012). Especially, it has only two class II heme-peroxidase, i.e. MnP, encoding genes. However, as mentioned before, *A. bisporus* possesses a highly expanded set of putative HTP encoding genes, including chloroperoxidases and aromatic peroxygenases. Although the exact biological role of these enzymes is not understood (Hofrichter et al. 2015), they are likely related to the conversion of high content of humic compounds in the natural habitats of *A. bisporus*.

Research performed on compost composition during mycelium and mushroom growth is difficult to compare. The variability in raw ingredients is large, and different composting conditions, e.g. length of composting phases, are in place. Nevertheless, changes in wheat straw-based compost and its structural components were studied previously, and mainly total carbohydrate content, and lignin and inorganic constituents were investigated on a relative base (Gerrits et al. 1967; Iiyama et al. 1994; Lyons et al. 2006). Compost samples were collected at different phases, and although composting conditions were not the same, the obtained results suggest loss of dry matter during composting, and carbohydrate degradation during composting and overall fungal growth (Iiyama et al. 1994; Durrant et al. 1991). Further, the degradation of ^14^C-labelled lignin indicates that lignin is mainly degraded during mycelial growth (Wood and Leatham 1983).

### Reproduction phase of *A. bisporus*—changes in plant cell wall structures

During the reproductive fruiting body formation phases of *A. bisporus* (Filling, Pinning, first flush, and second flush; Fig. 2), it is found that in addition to carbohydrate consumption, also substituted xylan accumulates. The degree of xylan (X) substitution with arabinosyl (A)- and (4-*O*-methyl-)glucuronic acid (UA) of from compost extracted xylan increased for one xylan population from 0.11 to 0.16 for A/X and from 0.10 to 0.17 for UA/X (Jurak et al. 2015b, c). For a second xylan population, A/X increased from 0.14 to 0.21 and UA/X from 0.15 to 0.28 (Jurak et al. 2015b, c). These observed increases correlated well with the observed gene expression and enzyme activities. Although the *A. bisporus* genes encoding putative family GH43 enzymes were expressed during the first flush in compost (Patyshakuliyeva et al. 2013), phylogenetical analysis showed that these enzymes are not likely to be active towards the doubly substituted xylan (Jurak et al. 2015c). The later study also demonstrated that no arabinofuranosidase activity able to release arabinosyl units from doubly substituted xylooligomers was found in compost extracts. Also, in the *A. bisporus* genome, two genes encoding putative α-glucuronidases are present, but these are not significantly expressed in compost and their corresponding activity was also not detected in the compost (Jurak et al. 2015b).

It has to be remarked that compositional changes in xylan structure, as explained above, do not reflect changes in absolute amounts of carbohydrates in the compost during fruiting body formation. The quantification of the decrease of carbohydrates, however, can be calculated based on decreases in total dry matter of compost (Baars and Sonnenberg 2014). By using the carbohydrate content values (% *w*/*w*) obtained (Jurak et al. 2015c), the mass balance of total carbohydrates, xylan and cellulose, was calculated (Table 1). During the fruiting body formation phase, 40% of total carbohydrates from the compost was consumed. In more detail, xylan was consumed to a larger extent (50%) compared to glucan (35%) (Table 1). During mushroom production, endoxylanase, β-xylosidase, endoglucanase and β-glucanase activities were detected in the compost extracts (Jurak et al. 2015b). Unfortunately, glucan in compost is present as both cellulose and mycelial cell wall glucan. Hence, the consumption of specifically cellulose or mycelial cell wall glucan could not be distinguished.

**Table 1:** Dry matter, total carbohydrates, xylan and glucan content (% *w*/w based on dry matter (DM)) and mass balance of compost from end of PIII to second flush

	Content (*w*/*w*% DM)		Mass balance (kg/m^2^)			Loss
	End PIII^a^	Second flush^a^	End PIII^b^	Second flush^b^	Loss	Relative to end PIII
Dry matter	37	39	30.9	25.9	5	16%
Total carbohydrates	21.9	15.7	6.8	4.1	2.7	40%
Glucan	10.4	8.1	3.2	2.1	1.1	35%
Xylan	9.3	5.5	2.9	1.4	1.5	50%

^a^Values based on Jurak et al. (2015b)

^b^Based on report of Baars and Sonnenberg (2014)

Transcript levels (Patyshakuliyeva et al. 2013) and enzyme activities detected from the compost extracts (Jurak et al. 2015b, c) showed that the xylan depolymerizing machinery of *A. bisporus* was active throughout the cultivation in compost, and high xylan hydrolysing activity was present especially during fruiting body formation. The lowest xylanolytic activity was detected at the start of the cultivation process (at ‘Filling’), but it increased during the cultivation process resulting in the highest activity after harvesting the first flush and then decreased by 20% until harvesting the second flush (Jurak et al. 2015b). This is in good agreement with the xylan content of the compost analysed in various stages of the composting.

Despite the upregulation of pectinase and cellulose encoding genes during the first flush (Patyshakuliyeva et al. 2013), no homogalacturonan, rhamnogalacturonan I (RGI) or CMC-related enzyme activities were detected at this phase. While it remains unclear whether these pectin degrading enzymes are produced, the lack of CMC-related enzyme activities in the spawning stage day 16 may be due to the suggested connection between cellulase production and fruiting body development for *A. bisporus* (Claydon et al. 1988).

Possibly reflecting the higher need for carbon during the fruiting body formation, polysaccharide degrading enzyme encoding genes were mainly expressed after the spawning stage, and their transcript levels peaked during the first flush and after the second flush (Patyshakuliyeva et al. 2015). Both xylanase and cellulose encoding genes were expressed, indicating that these polysaccharides are degraded simultaneously by *A. bisporus*. The reduction in the transcript amounts after harvesting of the first flush mushrooms was not fully reflected by the proteome, since cellulolytic CAZymes were detected, most likely due to stability of the proteins.

The most abundant cellulases in compost were cellobiohydrolases (GH6 and GH7), endoglucanase (GH5_5) and β-glucosidase (GH3), whereas endoxylanases (GH10 and GH11), α-galactosidase (GH27), α-xylosidase (GH31), α-glucosidase, β-galactosidase (GH35) and α-arabinofuranosidase (GH51) represented the highly secreted hemicellulases (Patyshakuliyeva et al. 2015).

## Outlook on how molecular and enzyme insights together with mapping of substrate alterations can help to improve mushroom production

### Methodology

Further understanding on how mushrooms are produced on carbohydrates and lignin is still needed. In this review, it is shown that ‘top to bottom’ analysis of carbohydrates is a good approach to indicate changes in carbohydrates. At the level of total carbohydrate content, a ‘screening’ is obtained, while fractionation of the compost populations provides more details on the structures remaining.

Although in this review, structural changes are highlighted, corresponding to *A. bisporus* gene expression and enzyme activities, current methodologies are not able to (i) distinguish well between compost carbohydrates and microbial carbohydrates, (ii) measure amounts of chitin as a measure for extent of *A. bisporus* colonization and (iii) differentiate in detail which and how much of the lignin-like structures are metabolized. Further advancements on these three challenges will help in further unravelling the complex system of *A. bisporus* growth on compost.

### Improvements of *A. bisporus* colonization and fruiting

During mycelium growth, lignin-/humic-like substances are degraded, which is proposed to help the accessibility of carbohydrates in the compost during fruiting body formation. Furthermore, a compost which is colonized to a further extent with mycelium is known to generate more fruiting bodies in the following growth phase. Either a faster mycelium growth or further colonization, while at the same time lignin-/humic-like substances are metabolized, may become a reality by using an improved *A. bisporus* strain. Such a selective, faster growing strain, may result from careful strain selection, breeding or from genetic modification approaches. Selection criteria should be the ability to metabolize lignin-/humic-like structures during mycelium growth and dense colonization abilities, while maintaining the ability to produce commercial quantities of fruiting bodies in the reproductive phase.
